# Recent Developments in Inorganic Composites in Rotational Molding

**DOI:** 10.3390/polym14235260

**Published:** 2022-12-02

**Authors:** Zaida Ortega, Mark McCourt, Francisco Romero, Luis Suárez, Eoin Cunningham

**Affiliations:** 1 Departamento de Ingeniería de Procesos, Universidad de Las Palmas de Gran Canaria, 35017 Las Palmas de Gran Canaria, Spain; 2 Polymer Processing Research Centre, School of Mechanical and Aerospace Engineering, University of Belfast, Belfast BT9 5AH, UK; 3 Departamento de Ingeniería Mecánica, Universidad de Las Palmas de Gran Canaria, 35017 Las Palmas, Spain; 4 School of Mechanical and Aerospace Engineering, University of Belfast, Belfast BT9 5AH, UK

**Keywords:** rotational molding, composite, filler, reinforcement, fiber, waste, inorganic, mineral

## Abstract

Rotational molding allows for obtaining hollow parts with good aesthetics and properties, having as main drawbacks the lack of pressure and the long cycle times, which limit the range of materials. Different fillers have been introduced in rotomolding to obtain composite materials assessed. This review has shown that glass fibers or particles are the most common material among them, although carbon fibers or clays have also been studied. In general terms, 10% loadings provide an increase in mechanical properties; higher loadings usually lead to a decrease in processability or final properties. When the filler consists of a micro- or nano-material, such as clay or graphene, lower loadings are proposed, generally not exceeding 3%. The use of fillers of an inorganic nature to obtain composites has not been as explored as the incorporation of lignocellulosic materials and even less if referring to waste materials or side streams from industrial processes. So, there is a broad field for assessing the processing and properties of rotomolded composites containing inorganic waste materials, including the study of the relationship between the ratio of filler/reinforcement and the final properties and also their preprocessing (dry blending vs. melting compounding).

## 1. Introduction

Rotational molding allows for obtaining hollow parts with good mechanical properties and surface quality, even though no pressure is applied in the process. Some of the main drawbacks of this technology are the long cycle times, the relative difficulty in obtaining complex geometries, and the lack of a wide range of materials. Polyethylene (PE) accounts for more than 80% of the rotomolding market [1], with polylactic acid (PLA) as the second most used thermoplastic. Very few materials are used in rotational molding due to the process’s particularities and sensitiveness. Several attempts have been made to introduce recycled resources in rotomolded parts as a way not only to increase the range of materials available but also to advance through the development of more sustainable solutions. Some authors have tried rotomolding recycled PLA fractions, where it is vital to control the process accurately to reduce polymer degradation [2] or post-consumer plastic residues [3]. The last study highlights the difficulties of obtaining relatively pure fractions of the PE, leading to the obtaining of parts with reduced mechanical properties. To overcome this, Arribasplata-Seguin et al. have used recycled PE from bottlecaps blended with waste wood powder [4]. Another possible path is using blended recycled/virgin materials, which has also been proposed and achieved satisfactory results for 50/50 blends [5]. Finally, Dou and Rodrigue have proposed using HDPE from recycled bottles to obtain foamed parts [6]. 

Another strategy to advance toward more sustainable solutions is using residues or natural fibers, which increases the part’s mechanical properties while also reducing the amount of virgin plastic used in the process. In this sense, some authors have proposed using cable wastes [7], or recycled rubber [8,9]. Lignocellulosic fibers, such as banana or abaca [10], and agave [11], have been introduced in rotomolded parts at different loadings, generally not exceeding 10%. Some other works have increased this amount when using specific particle size distribution of the foreign material [12] or compatibilizers [13,14]. In any case, using 10% filler (in weight) is relatively common in rotational molding. 

Alternatively, the use of inorganic fillers, such as clays [15,16], quartz [17], halloysite [18], glass fibers or powder [19,20,21], copper slag [22], or graphite [23,24] has also been explored. This review focuses on the use of these types of materials in rotational molding, with particular attention to those coming from waste or natural origin. Lignocellulosic fillers in rotational molding have been recently reviewed [25]. So, they are excluded from this work, although some of them also come from waste resources, such as wheat bran [26], banana fibers [10], buckwheat husks [12], or wood dust [27]. 

## 2. Materials and Methods

The literature review was performed using Scopus and Web of Science search engines, with the keywords listed in Table 1, which also shows the number of works found for each search in both databases (searches performed in late October 2022). Apart from the terms listed below, “rotational molding” or “rotomolding” were also used only to retrieve the works related to this processing technology. The search using only “rotational molding” led to 1034 publications in Scopus and 388 in WoS, while “rotomolding” led to 207 and 112, respectively. Despite these two databases being within the bigger databases and having a considerable number of references, the differences between them in the searches performed are apparent. Scopus can retrieve a higher number of documents than WoS.

On the other hand, it also seems that most authors use “rotational molding” instead of rotomolding; if both terms are combined, 1103 results are found in Scopus and 446 in WoS, that is, there are some papers where both terms are used. It is also interesting to notice that a significant proportion of documents found in these searches relate to composites, which shows the great interest in such materials for this process (most of the papers from WoS and over 40% of Scopus results, in fact, contain the term composite). However, when the term “cellulose” is excluded, thus removing those studies dealing with lignocellulosic materials, the number of documents substantially decreases (especially for Scopus). This also demonstrates the relatively low intensity of research performed in the area of non-cellulosic fillers and the potential development in this area. When introducing “waste” or “residue” in the search, the number of documents is also reduced, but it can still be considered relevant, which demonstrates the interest of the sector in advancing toward the revalorization of such materials.

**Figure 1:**
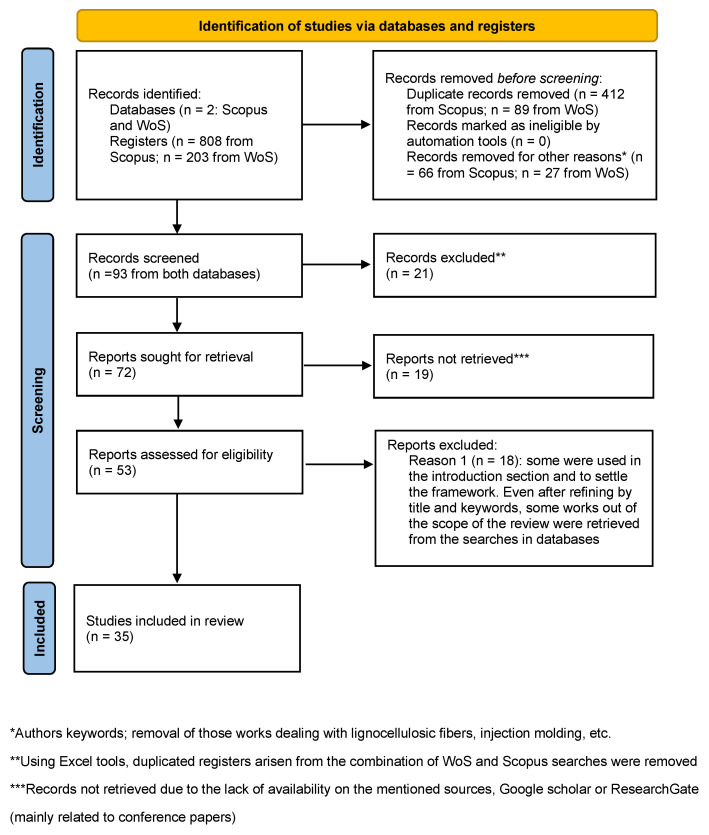
Flow diagram for the literature search.

Once an initial overview of the studies performed was obtained, and considering the focus of this paper, details from combined searches in both databases highlighted in Table 1 were downloaded and organized in the form of a table (these were found using the combination of “rotational molding” and “rotomolding” to ensure all documents appeared in the search). All elements were compared for each database and primary keyword (rotational molding or rotomolding), and repeated elements were removed. The data from 808 references were downloaded from Scopus and 203 from Web of Science; after eliminating duplicates, each list’s total number of works was reduced to 412 and 89, respectively. Even if the search was done including in all cases the terms closer to the aim of this work, the retrieved documents provided results out of scope, including lignocellulosic composites (wood flour, agave, or flax, among others), or other processing technologies (injection molding or casting). To remove such documents, authors’ keywords were analyzed, and only those papers related to the topic were kept. From the Scopus search, the list was reduced to 66 documents; from WoS, finally, 27 documents were listed. After combining them and again removing duplicates, 72 works were determined. Finally, to obtain an updated state of the art on this topic, only those works from the last years (from 2010, in particular) are retrieved, thus getting 35 papers as the core of this study. These led to 360 keywords in total, which were grouped by similarity. A flow diagram, following PRISMA guidelines summarizing the process for the literature search, is found in Figure 1.

**Figure 2:**
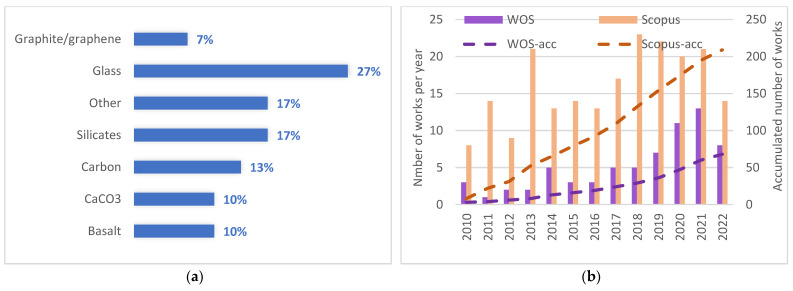
(**a**) Distribution of keywords related to materials from the literature review, (**b**) evolution of published papers dealing with rotomolded composites since 2010 (left axis: works published each year, in columns; right axis: accumulated works, in lines).

If analyzing the fillers or reinforcements, glass and carbon fibers are the most common ones (6 works on glass fibers or particles, and 4 on carbon fibers), followed by calcium carbonate, basalt, and carbon black. Graphite, silica, copper slag, talc, or graphite are other materials appearing, interestingly related to micro and nanocomposites. Figure 2a shows the most usual inorganic materials in rotational molding; as glass fibers are the most significant group, they have been separated from the “silicates” group, which includes halloysite, talc, or vermiculite. The category “Others” refers to carbon black (carbon relates to carbon fibers) and titanium and zinc oxides. Figure 2b shows the evolution of papers published in both sources (Scopus and WoS), showing an increasing trend in the number of works published in the field of rotational molding and inorganic fillers or reinforcements. 

## 3. Analysis of the Retrieved Works

The works retrieved from the literature search have been assessed and organized by type of filler, considering the materials with the most significance (by the number of published papers). So, three subsections have been considered: glass-derived materials, nano- or micro-sized fillers, and other materials. 

In most works, the different materials used for the composite obtaining are dry blended, although some propose using melt compounding. Dry blending consists of a mechanical mix of the polymer and the filler/reinforcement, usually in high-speed mixers. This method requires that the polymer is in powder form, which might lead to final parts with high porosity [16]. On the other hand, melt compounding allows for obtaining a more homogeneous material, although with higher energy requirements and possible oxidation or thermal degradation [16,20]. 

From the works retrieved from the literature search, it is not possible to perform a direct comparison between both methods of materials preparation, as composites are obtained using different matrices and fillers, sometimes treated, and in different ratios. However, Anisko and collaborators have performed a comparison between both methods, using vermiculite particles (lower than 200 μm) in loadings up to 10% as filler of an HDPE matrix. The rotomolding process was performed in similar conditions, and the final parts’ porosity, density, and mechanical properties were analyzed. These authors have found that compounding provided better properties (Table 2) and lower porosity and explained these findings by the geometry of the filler (flakes with irregular shapes). Similarly, Mhike and Focke [23] compared the behavior of LLDPE/graphite composites obtained by dry-blending and melt compounding, arriving at similar conclusions: better homogeneity of the final part, leading to lower variability, although not always to better properties due to thermal degradation. In any case, compounding is especially recommended for higher loadings (over 10% typically) or small particle sizes [24], and it is sometimes the only way to increase the ratio of filler; otherwise, depending on the filler density, parts are not consolidated. In any case, it is interesting to highlight that this last work used an antioxidant during the compounding process to reduce the thermal degradation of the materials. 

In the papers assessed, compounding is usually employed for fillers with lower particle sizes (under 20 μm), while dry blending is mainly used for fibrous fillers (high aspect ratio) or bigger particle sizes. However, some authors also employ dry blending for nano-sized particles, such as Zepeda-Rodríguez and collaborators [28], who obtained composites with carbon nanofibers (with diameters under 150 nm) by dry blending in a high shear mixing, or Shirinbayan et al. [29], who performed the blending by simple shaking of the PA and the nanocarbon black.

**Table 2:** Summary of results obtained from the literature for composites with inorganic fillers and reinforcements and several polymer composites (σ: strength, E: elastic modulus, both given as variation regarding the neat polymer matrix and given in %).

Filler	Size	Density (g·cm^−3^)	Ratio (%)	Preprocessing	Treatment	Matrix	Tensile		Flexural		Impact	Ref.
							σ	E	σ	E		
Vermiculite	<200 μm	--	10	Dry blending	No	HDPE	−76	−40	−63	35	−66	[16]
				Molten blending			−14	18	45	−50	−54	
Quartz flour	<100 μm	2.65	5	Dry blending	PE-MA	LLDPE	−23	0	--	--	--	[17]
Glass dust	150 μm	2.56	20	Dry blending	No	LLDPE	7	41	--	--	21	[19]
Graphite	100–500 μm	2.26		Dry blending	Antioxidant	LLDPE	−35	0	--	--	−87	[23]
				Molten blending			−43	−12	--	--	−87	
Glass fiber	14 μm ^1^	--	20	Dry blending	Plasma	LLDPE	20	20	--	99	−62	[21]
Hollow glass microspheres	20 μm	0.46	20	Melt blending	Hydroxylation + silanization	PE	1	49	--	--	--	[30]
						PCL	8	110	--	--	--	[31]
Copper slag	30–500 μm	3.04	10	Dry blending	No	PLA	−20	21	--	--	0	[22]
Basalt powder	10 μm	--	20	Mixing	No	PU	13	33	--	--	--	[32]
Halloysite nanotubes	<4 μm	2.55	5	Molten blending	No	HDPE	−23	35	0	0	−64	[33]
						LMDPE	−8	0	0	9	−54	
CaCO_3_	2 μm	--	10	Molten blending	Slip agent (erucamide)	LMDPE	0	−39	--	--	−35	[34]
TiO_2_/ZnO	Nano	--	2	Molten blending	No	HDPE	10	--	--	15	--	[35]
Carbon black	Nano	0.1	0.5	Dry blending	No	PA-12	20	0	--	--	--	[29]
Carbon nanofiber	Nano	--	0.1	Dry blending	Plasma	LMDPE	10	17	--	--	20	[28]
Graphite	Nano	2.26	0.25	Dry blending	Sonication + antioxidant	LLDPE	--	--	--	--	−80	[24]
				Molten blending			−10	--	--	--	−31	
Fumed silica	Nano	--	4	Molten blending	No	LLDPE	10	150	--	18	−7	[36]

^1^ Fibers with 0.19 mm length.

The assessment in more detail of the preprocessing conditions of the different materials before rotomolding them is an interesting path in which various aspects should be considered: homogeneity of the final material, porosity, properties, energy consumption, availability of materials, etc.

An exciting gap is then identified here: how does preprocessing of materials and their blend affect the final properties of the rotomolded parts? Does the introduction of a higher amount of filler or the better properties obtained justify the a priori higher energy consumption from melt compounding?

Table 2 summarizes the compositions of several composites from this literature review and the modifications found in mechanical properties, expressing the results as variations (in percentage) regarding the neat matrix processed in the same conditions. As observed, the most common matrix is polyethylene, and linear low-density polyethylene (LLDPE) in particular. Different behavior is observed depending on the type of filler used, the ratio of fiber to polymer, and the preprocessing (treatment or not of the fiber or the polymer, and blending of both components). Glass fibers treated with plasma improve the tensile and flexural behavior of the LLDPE, while a drastic decrease in impact properties arises. The same material (glass) in different configuration (powder), without any treatment, and at the same loading of 20%, also leads to higher tensile properties, although less significant, but with a further increase in impact strength, probably due to the shape of the particles. 

It can be seen that nano-scale fillers are used in lower proportions and that compounding is usually applied to improve the particle distribution in the matrix. It is also observed that most works report a significant decrease in impact properties and an increase in moduli; strength usually increases, although reductions are also reported in some works. 

It is also interesting to note that impact properties are generally reduced, despite the type of filler, properties, or processing. This highlights the high sensitivity of rotomolding to the introduction of foreign materials due to the lack of internal pressure. This fact is one of the main limitations of the process and has slowed the adoption of new materials within this sector. 

### 3.1. Composites with Glass Fibers or Glass Particles

Glass fiber is one of the most studied reinforcements in polymer composites and has also been studied in rotational molding. The control of the process, especially the densification stage, has been proved relevant for optimizing the final part [37], as the introduction of foreign materials can increase the number of bubbles or voids, thus decreasing the mechanical properties [38]. For example, Ghanem et al. [21] have obtained composites with up to 20% of glass fibers, studying the effect of plasma treatment on the matrix and the fibers. They found that treating both materials led to an increase in mechanical properties. However, the use of plasma at a large scale is still not thoroughly addressed, as the modifications achieved usually last only for a short period of time. Elastic moduli (tensile and flexural) and tensile strength rise with the increase in fiber content, while impact properties show the opposite trend. Gupta and Ramkumar introduced glass fibers in LLDPE [39], finding that 20% provided optimum rheological properties for the later rotomolding of the composite. These same authors later used glass fiber dust, a residue from wires insulation, into an LDPE matrix at 20% loading, blending both materials in a high-speed mixer to reduce the thermal degradation from the molten compounding, obtaining moderate increases in mechanical properties, even in impact strength [19]. Glass particles have also been proposed as reinforcement of PE in up to 20% [30]; this research highlights the importance of using a coupling agent, such as maleic anhydride grafted polyethylene, to improve interfacial bonding and ultimately the composite’s performance. The addition of such a compound results in higher increases in elastic modulus and avoids the decrease in tensile strength observed for composites without that compound.

Vignali et al. [31] have incorporated glass hollow microspheres into a PCL matrix, obtaining a composite with higher thermal stability than the matrix. They have found that the treatment of the glass particles is needed to increase the tensile modulus of composites and to avoid a reduction in tensile strength. The surface treatment proposed by these authors consisted of a first step of hydroxylation with NaOH, followed by a later silanization, as also presented by Stagnaro for PE-based composites [30]. 

From Table 2, the highest tensile moduli increase is found for composites with glass particles (49 and 41% for hollow glass microspheres and glass dust, respectively, in a PE matrix) and fumed silica (an increase of 150%, also in PE), at 20% loading for glass and only 4% for silica (nano-sized). It is interesting to note that glass dust seems to provide higher improvements in tensile properties and does not decrease impact strength, compared to glass fibers, maybe due to the particle size used. In any case, comparing the results from the different works is not easy, as other processing conditions were applied, and the properties assessed do not always coincide.

### 3.2. Composites with Nanoparticles

Nanopowders have also been used in PE matrices by rotomolding. Ghanbarpour and Moslemi [35], for example, incorporated up to 2% of a nanopowder consisting of titanium and zinc oxides (TiO_2_/ZnO, in a 50/50 proportion), increasing flexural moduli, although not finding any other differences in mechanical properties. However, the composite provided interesting antibacterial properties. 

Daryadel et al. [15] introduced nanoclay at 2% into an LLDPE matrix, obtaining an increase in tensile properties when optimizing cycle conditions. For these loading levels, these authors have proved more efficient optimization of process parameters (temperature and rotation ratios) than introducing a potential reinforcement. Finally, oxygen-treated plasma carbon nanofibers have been successfully introduced in a PE matrix with significant increases in mechanical properties, especially in toughness and impact strength [28], when used at very low loadings of 0.01% or 0.1%. An increased amount of such fibers led to the reduction of properties. Sangeetha et al. proposed using halloysite nanotubes for rotomolding, but they performed the experimental work with compression molding [18]; after the obtained results, these authors considered that proportions up to 10% in LLDPE would provide better mechanical properties and thermal stability. This material was used by Höfler and collaborators [33] to reinforce LMDPE and HDPE in various ratios. These authors have found better mechanical properties when the clay is compounded with the polymer; 5% of the halloysite resulted in an increase in the tensile modulus, although reducing the tensile strength. Improved barrier properties can also be obtained for nanoclay/PE rotomolded composites at these proportions [40]. 

Calò et al. [41] produced a composite with 5% of montmorillonite and a halide-based additive to obtain a composite with improved flame retardancy behavior. This last compound is needed to improve the thermal stability of the blend and make it rotomoldable. The properties of the matrix are not given in this work, although the authors state that elastic modulus is increased and tensile strength is reduced, as otherwise observed in other works. Interestingly, the heat release rate (flame retardancy) improved by a factor of 3 with the incorporation of the modified clay. 

Chandran and Waigonkar have performed several works with fumed silica in PE matrices [36,42,43,44]. By previously compounding both materials, they found significant improvements in tensile modulus with the addition of 4% silica and more discrete increases in tensile strength and flexural modulus (10 and 18%, respectively), with only a slight decrease in impact strength. Loadings over 4% resulted in lower mechanical properties of the composites due to the poor distribution of the silica in the matrix for such ratios. Even the melt compounding of both materials did not allow for the incorporation of higher amounts of filler homogeneously. 

Different types of graphite were also rotomolded in low loadings, evidencing the need to compound the graphite with the PE before the rotomolding. In these studies, authors obtained rotomolded parts with antistatic and fire-retardant properties [23,24], although with reduced mechanical properties, using the same matrix and expandable graphite at different ratios and processing. In [23], composites with up to 20% graphite were obtained, while in [24], the authors reduced the loadings and proposed a maximum use of 2%. Another significant difference between these two works is the graphite size. In the first work, larger particle sizes are used (from approximately 100 to 500 μm), while the second work focuses on nanocomposites and uses graphite nanoplatelets with an average size of 13 μm.

Nanocarbon black can improve the tensile properties of a PA-12 matrix only when used up to 0.5%; higher loadings lead to reduced mechanical properties [29]. 

Finally, Karimzadeh et al. have also explored the use of nanoclay and microtalc in foamed PE, obtaining improvements in cell density and size [45]; other properties were not discussed in that paper. 

### 3.3. Composites with Other Fillers

Głogowska et al. [17] obtained LLDPE-rotomolded composites with up to 35% quartz flour, using maleic anhydride-modified HDPE as a compatibilizer. This study did not notice any improvement in mechanical properties. Tensile strength and elastic modulus decreased when increasing the filler content, even with the use of the MAPE, although in a lower proportion; in fact, the modulus was not reduced for such samples. The surface roughness increased with the filler content. 

Baumer et al. [34] have incorporated up to 10% of calcium carbonate powder in LMDPE, finding increased values of porosity and thus a decrease in mechanical properties; furthermore, despite starting from a masterbatch of PE/CaCO_3_ and performing a compound with the PE to obtain the desired ratios of filler, a lack of adhesion with the PE is observed from SEM micrographs and so a coupling agent or the modification of the filler would be needed. 

Barczewski et al. [22] have studied the introduction of copper slag, a residue from mining activity, in a PLA matrix, improving composite stiffness with up to 20% loading. All composites with this filler showed reduced tensile strength, especially over 10%; surprisingly, impact properties were not modified by adding the copper slag up to 10%. This same team proposed using basalt powder as a filler of polyurethane in reactive rotational molding [32], finding increased properties (tensile strength and modulus and hardness) with basalt loading of up to 20%, although obtaining lower homogeneity in wall thickness due to the formation of a foamed structure. 

Finally, some authors have introduced the filler directly in the polymerization stage. For example, Rusu has performed in situ polymerization of nylon 612 with TiO_2_ particles in 8%, obtaining a higher value for glass transition temperature and improvements in flexural properties of approximately 13% and 30% for strength and moduli, respectively [46]. 

Waste materials from the mining industry can be of particular interest to the rotomolding sector, as they are usually found in the form of thin powders, are of inorganic nature (thus not being especially sensitive to thermal degradations), and are produced in large amounts yearly and worldwide. In addition, the accumulation of such residues usually pose an environmental problem. For example, red mud is obtained from the aluminum extraction process in a proportion of 2.5–4 tons of red mug per ton of aluminum; copper slag is produced in a similar proportion in the copper obtaining process (approximately 2 tons/ton of copper) [22]. Quarries also yield a significant amount of mineral powder residues; for example, only in the Apuan Alps district, over 200,000 tons of marble dust are produced yearly, and similar values can be found for other mineral quarries, such as basalt stone [47,48]. 

## 4. Conclusions

Several materials can be introduced in the rotational molding process to produce composites with improved mechanical, thermal, aesthetics, or environmental properties. Most works dealing with rotomolded composites use polyethylene as a matrix, which is the most common material used for this manufacturing process. For composites production, lignocellulosic materials are the most studied ones. The works performed in this field also include the valorization of waste biomass or side products. 

Apart from these organic natural materials, other inorganic compounds can be introduced into this process, these having the advantage, in general, of having higher thermal stability. Among them, the most studied to date are hollow glass particles and glass fibers. Some other materials, such as carbon fibers or carbon black, have also been explored in the literature. The use of other inorganic materials, such as mineral dust or industrial processes side streams, still remain almost unexplored.

There is a growing interest in using waste or side materials in the polymer processing industry as a way to improve the properties of the final parts, including their environmental footprint and economic cost, while also impacting the variety of materials available for rotomolding and widening the range of properties achieved. 

Future works should address the optimal preprocessing of the materials (dry-blending or melt compounding), including the assessment of energy consumption and the environmental performance of final composites, as this is an almost unexplored field to date. 

On the other hand, there is still a promising field to develop in dealing with the valorization of wastes in the rotational molding industry. There is a wide range of waste materials or by-products, such as basalt, glass powder, red mug, etc., available in powder form and in massive amounts that could provide interesting properties to polymer-based composites. These include not only mechanical behavior but also flame retardancy, aesthetics, durability, and electric conductivity, among others.

The introduction of such materials, together with the implementation of lifecycle assessment tools and the improvement of the energy efficiency of the entire process, including preprocessing of materials, would significantly contribute to better environmental behavior of this technology and more sustainable products. The study in detail of the preprocessing would increase knowledge on the rotomolding process and how the materials behave inside the mold while heating and rotating at the same time, thus leading to understanding the relationship between filler distribution and the final parts’ properties, and in strategies to improve them from the early stages of materials preparation. 

Finally, further efforts are needed to advance toward more sustainable and responsible production processes and in the development of tools to accurately determine the environmental behavior of materials used in the different technologies and applications.

An exciting path is then open here to fill in the gap existing in rotomolding and the use of side streams of an inorganic nature as a way to increase the materials range availability, sustainable character of the technology and also as a way to obtain parts with improved functionalities.

## Figures and Tables

**Table 1:** Number of papers found from Scopus and WoS searches.

Keywords	Scopus	WoS
Composite	447	393
Inorganic	33	6
Inorganic + composite	23	5
Composite – cellulose ^1^	379	101
Composite + fiber	230	47
Composite + filler	86	22
Composite + fiber − cellulose	170	47
Composite + filler − cellulose	62	22
Waste or residue	106	18
Composite + waste (or residue)	78	6
Waste or residue – cellulose	86	17
Composite + waste (or residue) − cellulose	59	5

^1^ Cellulose, lignocellulose, cellulosic, lignocellulosic.

## Data Availability

Not applicable.
